# Malignant Mesothelioma Presenting as a Gradually Enlarging Pneumothorax

**DOI:** 10.1155/2013/374960

**Published:** 2013-10-02

**Authors:** Ashish Prasad, Diana Olsen, P. S. Sriram

**Affiliations:** ^1^Division of Pulmonary, Critical Care and Sleep Medicine, University of Florida, Gainesville, FL 32610-0225, USA; ^2^Division of Pulmonary, Critical Care and Sleep Medicine, NFSG VA Medical Center, 1600 SW Archer Road, 111A, Gainesville, FL 32608, USA

## Abstract

Malignant mesothelioma is an extremely aggressive tumor arising from the pleura with median survival of approximately 9–12 months. It can rarely present as a spontaneous pneumothorax. Less than 35 cases of malignant mesothelioma presenting as spontaneous pneumothorax have been reported in the literature. Pathology may show florid mesothelial hyperplasia. We herein report a case of mesothelioma presenting as a pneumothorax that gradually enlarged over a one-year period and also review the relevant literature.

A 69-year-old man was referred to pulmonary clinic for a gradually enlarging right-sided pneumothorax. A year and a half prior to clinic visit, the patient had experienced 3 days of vomiting associated with retching. This was followed by a nonproductive cough without fever or chest pain. A chest radiograph revealed a small right-sided pleural effusion. A chest CT scan confirmed a small effusion and also showed a small right lower lobe infiltrate. An esophageal perforation was ruled out. He was treated with a 1-week course of oral fluoroquinolone with resolution of symptoms. A follow-up chest CT scan 3 months later showed a small anterior right pneumothorax and improvement in right-sided effusion. The patient was asymptomatic at that time. The right sided pneumothorax continued to gradually increase in size on subsequent CT scans; however, he declined any workup given lack of symptoms. A few months prior to our evaluation, he started noticing gradually progressive exertional dyspnea and a nonproductive cough. He denied fevers, weight loss, chest pain, or hemoptysis. His past medical history was unremarkable except for glaucoma. He was a 75 pack year smoker who quit 3 years ago. He was exposed to asbestos while serving in the navy for 4 years and subsequently worked as a truck driver.

All physical findings were normal except for decreased breath sounds in the anterior right lower chest on auscultation. Blood count and biochemistry were normal. Serial chest CT scans over a 1-year period show gradual enlargement of the right sided pneumothorax. (Figures 1, 2, 3, and 4). The patient was referred to thoracic surgery and underwent posterolateral thoracotomy. He was noted to have densely adherent right lower lobe that was dissected free from the posterior chest wall. There was a cyst-like sac in the region of the bronchus intermedius, which was opened to reveal a bronchopleural fistula. The cyst was removed and the fistula was closed. Pathology of the resected specimen was initially read out as exuberant mesothelial hyperplasia. Further review revealed epithelioid cells arranged in tubules and irregular islands. On immunohistochemistry, the epithelioid cells were reactive to pancytokeratin, calretinin, D2-40, and keratin 5/6 confirming mesothelial origin. These cells were negative for CD15, CEA, and TTF-1. A final diagnosis of low-grade mesothelioma was made.

Malignant mesothelioma is an extremely aggressive tumor arising from the pleura causing nearly 20,000 deaths worldwide every year. Median survival is approximately 9–12 months. It is temporally associated with asbestos exposure, often occurring 30–40 years after exposure. There are 3 histological varieties of malignant mesothelioma that is epithelioid, sarcomatoid and biphasic, or mixed type. Mixed mesotheliomas have both epithelioid and sarcomatoid features. More than 50% of the cases are epithelioid, 10% are sarcomatoid, and the remainder are mixed types. It is often difficult histologically to distinguish mesothelioma from metastatic carcinomas, carcinosarcomas, and metastatic pleural sarcomas. Immunohistochemical markers, that is, pankeratin, keratin 5/6, calretinin, and Wilms Tumor-1 (WT-1), are useful to confirm mesothelioma while carcinoembryonic antigen, CD15 and thyroid transcription factor-1 (TTF-1) are negative markers [1].

Malignant mesothelioma rarely presents as a spontaneous pneumothorax. Less than 35 cases of malignant mesothelioma presenting as spontaneous pneumothorax have been reported in the literature. Most of them have been presented as rare case reports [2]. In one of the largest series of patients with recurrent or persistent spontaneous pneumothorax over a 5-year period who underwent pleurectomy, only 5 (4.3%) of the 91 cases were diagnosed with malignant mesothelioma. Malignant mesothelioma was not suspected on any of the patients during surgery. In one of the previous cases pleural histology was reported as florid mesothelial hyperplasia. Autopsy a year later showed extensive malignant mesothelioma. When the pleurectomy specimens were compared to the autopsy ones, it was felt that the florid mesothelial proliferation represented malignant mesothelioma. All 5 patients were above 40 years of age and 3 of them had known exposure to asbestos. Three patients had epithelioid mesothelioma and the remainder had the mixed type [3].

In another series of 4 patients presenting with spontaneous pneumothorax, one underwent thoracotomy, fistula resection, and surgical pleurodesis for recurrent pneumothorax. Pleural biopsy showed fibrosis. A year later, he presented with a large pleural mass consistent with epithelial mesothelioma. A second patient was noted to have nonspecific pleuritis on thoracotomy for recurrent pneumothorax. Nearly 2 years later, the patient presented with a large hydropneumothorax on the same side. Pleurectomy revealed epithelioid mesothelioma. Review of prior pathology was suggestive of mesothelioma. All 4 patients were above 55 years of age and had a remote exposure to asbestos. Three patients had epithelial mesothelioma and the 4th one had the sarcomatoid variety [4].

All 3 histological varieties, that is, epithelioid, sarcomatoid, and mixed type, can present as spontaneous pneumothorax. 

During surgery our patient had an entirely normal parietal pleural surface. We believe that the mesothelioma originated in the visceral pleura, and eroded inwards to cause a small bronchopleural fistula, which resulted in a gradually enlarging pneumothorax. Our patient decided against any further treatment including surgery and chemotherapy and died 3 years later due to disease progression.

In summary, patients with asbestos exposure, who present with recurrent or persistent spontaneous pneumothorax, should have the pleurectomy samples sent for histology to rule out malignant mesothelioma. If pleural pathology in patients with spontaneous pneumothorax who have a strong occupational exposure shows marked mesothelial hyperplasia, it is imperative to consider mesothelioma in the differential.

## Figures and Tables

**Figure 1:**
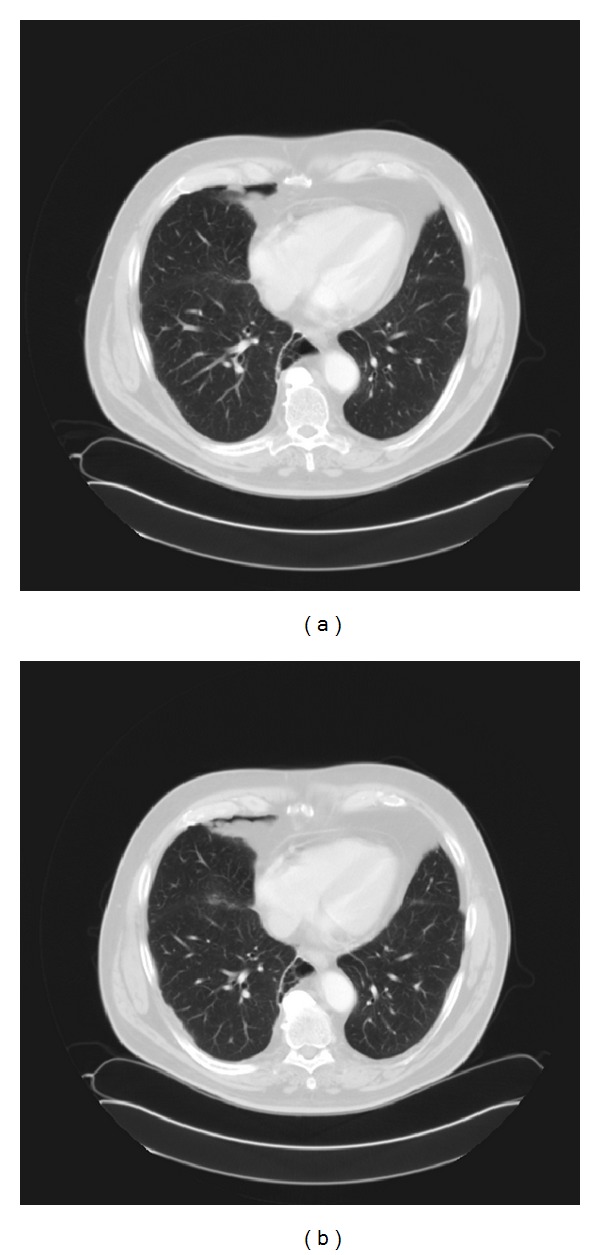
Chest CT scan in September 2007 showing a small anterior right pneumothorax.

**Figure 2:**
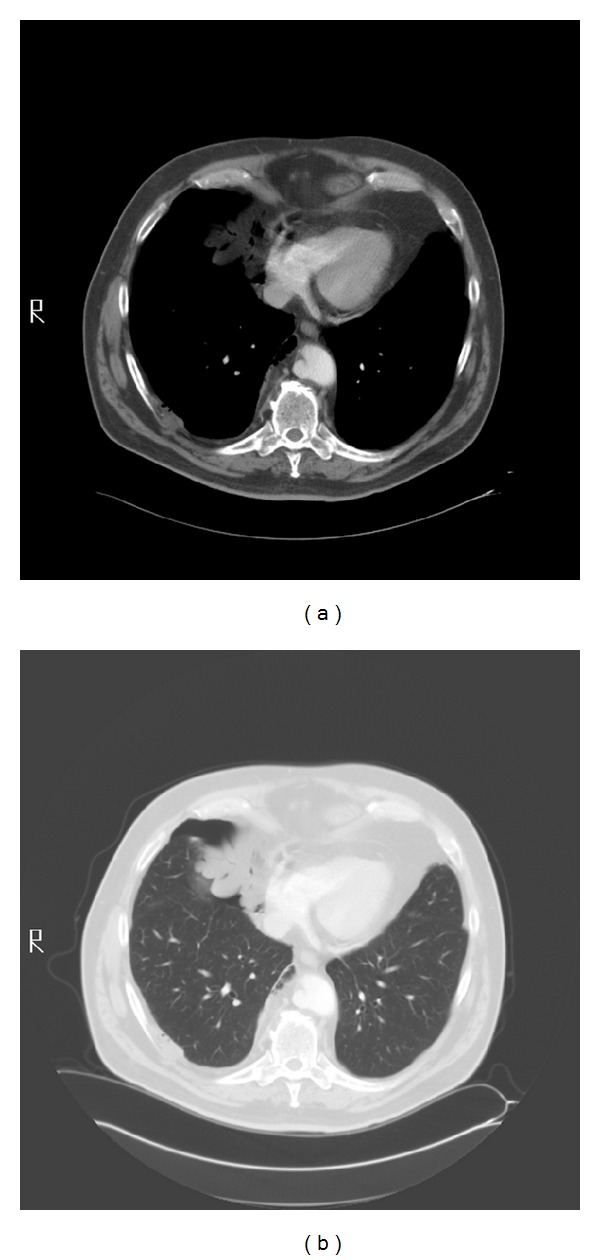
Chest CT scan in December 2007 showing a slightly larger anterior right pneumothorax. Corresponding soft tissue (mediastinal window view) showing that the soft-tissue mass adjacent to the pneumothorax is fat.

**Figure 3:**
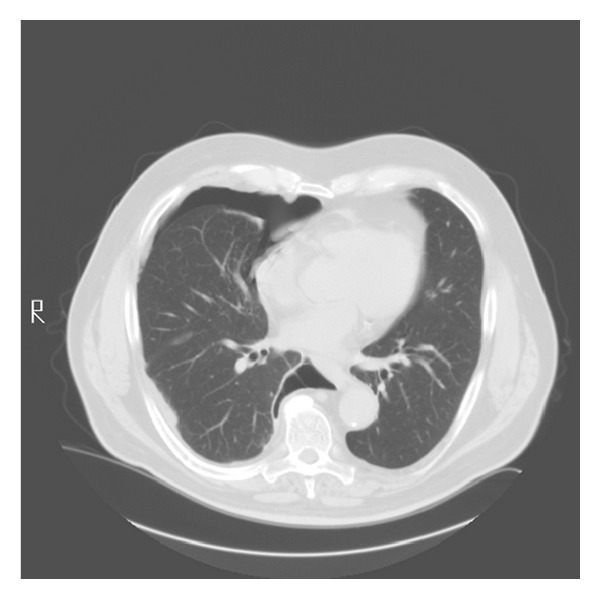
Chest CT scan in March 2008.

**Figure 4:**
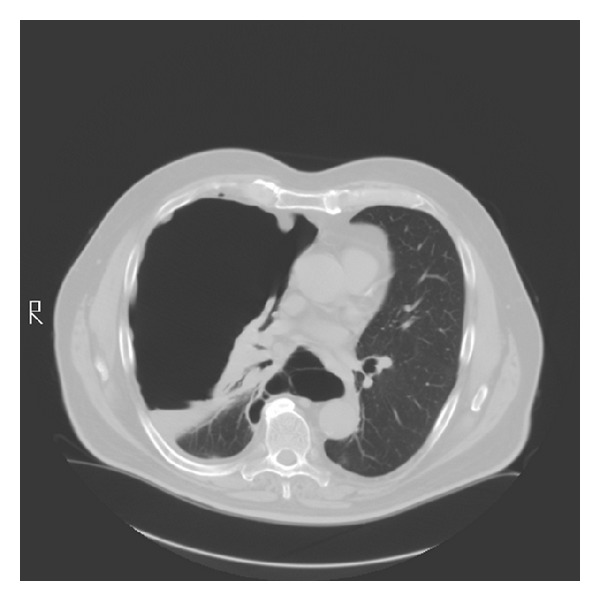
Chest CT scan in August 2008 showing a large right pneumothorax with atelectasis of the right upper and middle lobes.
